# Detection of *mecA* Genes in Hospital-Acquired MRSA and SOSA Strains Associated with Biofilm Formation

**DOI:** 10.3390/pathogens13030212

**Published:** 2024-02-28

**Authors:** Rosa González-Vázquez, María Guadalupe Córdova-Espinoza, Alejandro Escamilla-Gutiérrez, María del Rocío Herrera-Cuevas, Raquel González-Vázquez, Ana Laura Esquivel-Campos, Laura López-Pelcastre, Wendoline Torres-Cubillas, Lino Mayorga-Reyes, Felipe Mendoza-Pérez, María Angélica Gutiérrez-Nava, Silvia Giono-Cerezo

**Affiliations:** 1 Laboratorio de Bacteriologia Medica, Departamento de Microbiologia, Escuela Nacional de Ciencias Biologicas, Instituto Politecnico Nacional, Prolongacion de Carpio y Plan de Ayala S/N, Col. Casco de Santo Tomas, Alcaldia Miguel Hidalgo, Mexico City 11340, Mexico; mixtlipp@yahoo.com.mx (M.G.C.-E.); sgiono@yahoo.com (S.G.-C.); 2 Hospital de Especialidades, “Dr Antonio Fraga Mouret” Centro Medico Nacional La Raza, Instituto Mexicano del Seguro Social IMSS, Mexico City 02990, Mexico; laura.lopezp@imss.gob.mx (L.L.-P.); wendoline.torres@imss.gob.mx (W.T.-C.); 3 Laboratorio de Inmunologia, Escuela Militar de Graduados de Sanidad, Secretaria de la Defensa Nacional SEDENA, Mexico City 11200, Mexico; 4 Hospital General, “Dr Gaudencio Gonzalez Garza”, Centro Medico Nacional La Raza, Instituto Mexicano del Seguro Social IMSS, Mexico City 02990, Mexico; 5 Laboratorio de Biotecnologia, Departamento de Sistemas Biologicos, CONAHCYT-Universidad Autonoma Metropolitana Campus Xochimilco, Calzada del Hueso 1100, Col. Villa Quietud, Alcaldia Coyoacan, Mexico City 04960, Mexico; 6 Laboratorio de Biotecnologia, Departamento de Sistemas Biologicos, Universidad Autonoma Metropolitana Campus Xochimilco, Calzada del Hueso 1100, Col. Villa Quietud, Alcaldia Coyoacan, Mexico City 04960, Mexico; aesquivel@correo.xoc.uam.mx (A.L.E.-C.); lmayorga@correo.xoc.uam.mx (L.M.-R.); fmendoza@correo.xoc.uam.mx (F.M.-P.); 7 Laboratorio de Ecologia Microbiana, Departamento de Sistemas Biologicos, Universidad Autonoma Metropolitana Campus Xochimilco, Calzada del Hueso 1100, Col. Villa Quietud, Alcaldia Coyoacan, Mexico City 04960, Mexico; agutz@correo.xoc.uam.mx

**Keywords:** *mec*, healthcare-associated infections, biofilm, methicillin-resistant, *Staphylococcus aureus*, MRSA, SOSA

## Abstract

Methicillin-resistant (MR) *Staphylococcus aureus* (SA) and others, except for *Staphylococcus aureus* (SOSA), are common in healthcare-associated infections. SOSA encompass largely coagulase-negative staphylococci, including coagulase-positive staphylococcal species. Biofilm *formation* is encoded by the *icaADBC* operon and is involved in virulence. *mecA* encodes an additional penicillin-binding protein (PBP), PBP2a, that avoids the arrival of β-lactams at the target, found in the staphylococcal cassette chromosome *mec* (SCC*mec*). This work aims to detect *mecA*, the *bap* gene, the *icaADBC* operon, and types of SCC*mec* associated to biofilm in MRSA and SOSA strains. A total of 46% (37/80) of the strains were *S. aureus*, 44% (35/80) *S. epidermidis*, 5% (4/80) *S. haemolyticus*, 2.5% (2/80) *S. hominis*, 1.25% (1/80) *S. intermedius*, and 1.25% (1/80) *S. saprophyticus*. A total of 85% were MR, of which 95.5% showed *mecA* and 86.7% β-lactamase producers; thus, *Staphylococcus* may have more than one resistance mechanism. Healthcare-associated infection strains codified type I-III genes of SCC*mec*; types IV and V were associated to community-acquired strains (CA). Type II prevailed in MRSA *mecA* strains and type II and III in MRSOSA (methicillin-resistant staphylococci other than *Staphylococcus aureus*). The operon *icaADBC* was found in 24% of SA and 14% of SOSA; probably the arrangement of the operon, fork formation, and mutations influenced the variation. Methicillin resistance was mainly mediated by the *mecA* gene; however, there may be other mechanisms that also participate, since biofilm production is related to genes of the *icaADBC* operon and methicillin resistance was not associated with biofilm production. Therefore, it is necessary to strengthen surveillance to prevent the spread of these outbreaks both in the nosocomial environment and in the community.

## 1. Introduction

Due to hospital microbiota, the environment, antimicrobial-resistant bacteria, invasive procedures and devices, immunosuppression, age, comorbidity, and length of stay [1,2], healthcare-associated infections (HAIs) constitute one of the main public health problems that threaten the lives of sick or immunocompromised patients, increasing the costs of hospital care, as well as increasing antimicrobial resistance morbidity and mortality [3,4,5]. Bacteria of the ESKAPE group (*Enterococcus faecium*, *Staphylococcus aureus*, *Klebsiella pneumoniae*, *Acinetobacter baumannii*, *Pseudomonas aeruginosa*, *Escherichia coli*) have high diversity mechanisms of resistance to antimicrobials as an evolutionary strategy and due to adaptation and inadequate or excessive use of antibiotics [4]. Between 2016 and 2020, ESKAPE were the most isolated microorganisms in hospitals and were responsible for most HAIs with high virulence [4,6]. *S. aureus* is one of the main pathogens of both healthcare-associated and CA infections; because of their transmission routes, SCC*mec* typing, estimate prevalence, and antibiotic resistance, it is important to have a current epidemiological context [7,8]. MRSA infections have surged dramatically in the past 10–15 years and are increasingly becoming a major source of nosocomial infections that are associated with high morbidity and mortality. The incidence of MRSA infection ranges from 30 to 50 cases/100,000 population [9,10]; the range varies according to the development of the country and increases if the patient is immunocompromised. The 30 days in hospital mortality has been reported as 28.5% from a total of 221 patients [11].

Methicillin resistance in *S. aureus* is associated with the presence of the *mecA* gene that encodes the production of an unusual penicillin-binding protein (PBP), designated PBP2a, which weakens the affinity for β-lactam antibiotics [12]. A difference in biofilm formation by MRSA and methicillin-susceptible *S. aureus* (MSSA) has been suggested, but how only the presence of the SCC*mec* cassette or *mecA* influences this phenotype remains unclear. It has been reported that resistance to β-lactam antibiotics occurring in MRSA strains could be associated with the presence in the bacterial genome of transferable genomic islands, called SCC*mec*, where the *mec* gene determines resistance to methicillin. Within the different types of SCC*mec*, there may be *mecA* or *mecC* genes and resistance genes to other groups of antibiotics such as aminoglycosides, macrolides, lincosamides, streptogramins B, and tetracyclines [13].

However, the *icaADBC* operon is relevant in the PIA-dependent biofilms generated by MSSA. On the other hand, *bap* (biofilm-associated protein) is involved in the attachment to inert surfaces, intercellular adhesion, and biofilm formation and is a surface protein containing the LPXTG motif, which is responsible for the *ica*-independent biofilm formation in MRSA and MSSA strains [14]. Particularly, it is believed that *mecA* genes have been acquired from the *Staphylococcus sciuri* species group, which includes *S. fleurettii*, *S. lentus*, *S. sciuri*, *S. stepanovicci*, and *S. vitulinus*, found in soil, skin, and the mucous membranes of wild animals. *S. fleurettii* is an animal commensal bacterium that harbors the ancestral *mecA* gene, suggesting that MRSA probably acquired *mecA* from coagulase-negative staphylococci (SOSA) of animal origin. *mecA* and its new homologues (*mecB*, *mecC*, and *mecD*) share more than or equal to 70% nucleotide sequence similarity. The types designated reflect their chronological order of discovery: *mecA* was identified in *S. aureus* N315, *mecB* in *M. caseolyticus*, *mecC* in *S. aureus* LGA251, and *mecD* in *M. caseolyticus* IMD0819 [15]. The primary function of the original *mecA* gene was related to cell wall synthesis, but its evolution into a resistance determinant appears to have occurred via a stepwise process within the *S. sciuri* species group [16]. Particularly, *mecB* is flanked by *β*-lactam regulatory genes like *mecR*, *mecI*, and *blaZ* and is part of an 84.6-kb multidrug-resistance plasmid that harbors genes encoding additional resistances to aminoglycosides (aacA-aphD, aphA, and aadK) as well as macrolides (ermB) and tetracyclines (tetS) [17].

SOSA in animals is becoming more pathogenic increases antibiotic resistant and can potentially disseminate to humans [18]. In the U.S., mortality caused by MRSA remains the highest for any antibiotic-resistant pathogen, reported by the CDC to be at ~20,000 in 2018. Furthermore, there is increased recognition of the considerable clinical importance of methicillin-sensitive *S. aureus* strains. Some lineages such as sequence type (ST) 398 can have high virulence, causing fatal infections [19]. Thus, efforts have continued to evolve in preventing MRSA infections; it remains a major cause of increased mortality and morbidity, since infections caused by drug-resistant bacteria result in worse outcomes. Specific clones of MRSA are closely associated with virulence factors and drug susceptibility, and these trends are important as a basis for pathology, comorbidities, severity of infection, infectious disease care, treatment, and control. Particularly, the mortality rate due to *S. aureus* bacteremia is 20–30%, the mortality rate due to MRSA bacteremia is even higher at 20–50%, and the cure rate for MRSA infections is 50–60% [20]. In contrast to many other bacterial pathogens, which often rely on only one or a few toxins to promote disease, *S. aureus* produces an astounding array of virulent factors. These include a plethora of toxins and immune evasion factors and a vast array of protein and non-protein factors that enable host colonization during infection. While there has always been great interest in *S. aureus* virulence ever since this bacterium was first recognized as an important pathogen at the end of the 19th century, recent developments have increased research efforts into unraveling *S. aureus* virulence mechanisms [19].

Clones as ST5-1, ST5-II, ST36-II, ST45-II, and ST239 III of healthcare-associated MRSA can infect people in the hospital environment; however, CA-MRSA clones ST1-IV, ST5-IV, and ST8-IV [8] are the most representative from the community without predisposing risk factors [15]; these community-acquired infections by strains of MRSA (CA-MRSA) are genetically different from healthcare-associated MRSA (HA-MRSA). Unfortunately, CA-MRSA strains are multidrug-resistant and currently represent a hospital-acquired epidemic [21]. MRSA strains are resistant to β-lactams due to the acquisition of SCC*mec* that carries the *mecA* gene, which is responsible for methicillin resistance. 

Other allotypes associated with SCC*mec* have been found, including type I, II, III, IV, and V, depending on the nature of the *mec* and *ccr* gene complexes, which favors the transmission of methicillin resistance to strains acquired in the community [13,22]. Another element of the genome is the *icaADBC* operon that encodes proteins involved in biofilm formation, as well as the *bap* gene that encodes the Bap protein involved in intercellular adhesion, the accumulation of bacterial cells, and the establishment of biofilms on inert surfaces [23,24]. The positive correlation between *icaADBC* and biofilm production in a high percentage of *S. aureus* isolated from patients with burns has been reported [25], since products of the *ica* locus and polysaccharide intercellular adhesin (PIA) are critical for intercellular bacterial adherence and biofilm formation [26]. In fact, *icaA* and *icaD* are the main actors of PIA synthesis, and the enzymatic activity of *icaA* increases in the presence of *icaD*. Extensive persistence of *Staphylococcus* species in hospital environments, as nosocomial behavior, is associated with strains carrying *icaA* and *mecA* [27]. In addition, there are other potential alternative mechanisms that contribute to biofilm formation, such as the PIA-independent biofilm mechanism and the microbial surface component recognizing adhesive matrix molecules (MSCRAMMs), which in *S. aureus* are covalently linked to the cell wall by sortase via the LPXTG motif, and include the following proteins: clfA and clfB, fib, fnbA and fnbB, cna (collagen-binding protein), ebps, and eno (laminin-binding protein) [28]; other mechanisms, including several environmental factors, such as glucose, NaCl, and ethanol, influence biofilm elaboration by affecting *icaA* and *icaR* expression. For instance, expression of *icaA* was unaffected by ethanol directly; however, it increased by repressing *icaR* transcription. Conversely, the induction of *icaA* expression by glucose or NaCl was *icaR* independent [29]. Thus, the study of these genetic markers could further lead to the design of new drugs aimed at biofilm inhibition by inducing the activity of all or some *icaADBC* operon repressors. Nowadays, there are no antibiofilm drugs to combat *Staphylococcus* infections [30].

Because HAIs are a threat to public health, it is important to understand the involvement of genes and the antibiotic resistance of MRSA and SOSA involved in HAIs [31]. The aim of this work was to detect *mecA*, the *bap* gene, the *icaADBC* operon, and types of SCC*mec* associated with biofilm production in MRSA and MRSOSA biofilm-forming healthcare-associated strains. *icaADBC* gene detection could vary, probably due to the arrangement of the operon, fork formation, and mutations. Despite all strains being BP, not all amplified *icaADBC*; therefore, BP may be due to other mechanisms or genes not studied in this work.

## 2. Materials and Methods

### 2.1. Strain Isolation and Identification

The isolation of the strains was carried out in a public hospital in Mexico City, with different clinical origins (cerebrospinal fluid, catheter, blood culture, respiratory tract secretions, and urine). The strains were identified by colony, microscopic morphology, conventional biochemical, catalase, oxidase, and coagulase tests [32] and by using Vitek 2.0^®^ (Biomérieux, Lyon, France). This study did not involve humans [33].

### 2.2. Antibiotic Resistance Test of S. aureus Strains

Phenotypic MR was determined by the Kirby and Bauer method using cefoxitin (Ctx) 30 μg (BD BBL^®^ Sensi-Disc^®^ Antimicrobial discs) according to the CLSI (2016) document. Strains able to produce a halo equal to or higher than 22 mm were considered as sensitive to methicillin; values less than or equal to 21 mm were considered as resistant. The vancomycin profile was determined using Vitek 2.0^®^. Control strains were *S. aureus* ATCC 43330 resistant to cefoxitin, *S. aureus* ATCC 25923 sensitive to cefoxitin and vancomycin, and *S. aureus* USA300 resistant to cefoxitin and sensitive to vancomycin [34].

### 2.3. β-Lactamase Production

β-lactamase production was assessed using Cefinase^®^ discs (BD BBL^®^ Paper Disc) according to the manufacturer’s instructions. The formation of a pink color in the Cefinase^®^ discs indicated positive production of *β*-lactamase. *S. aureus* ATCC 29213 was used as a positive control [35].

### 2.4. Determination of Biofilm Production

To determine biofilm production, 96-well bales with 9.0 × 10^8^ CFU/mL of each *Staphylococcus* spp. were used in 100 μL of Müller–Hinton (MH) broth (Nunc MicroWell™). *S. aureus* ATCC 27543 at the same concentration was used as a positive control, and sterile MH broth was used as a negative control. The plates were incubated at 37 °C for 24 h.

To carry out the biofilm quantification, the culture medium was removed from the wells, and 100 μL of glutaraldehyde was added (Sunwise Chem Co., Shanghai, China) at 2.5% to each well and fixed for 1 min at room temperature. The excess was removed and washed with 100 μL of 1× PBS. Subsequently, each well was stained with crystal violet (CV) (Fisher Chemical, Pittsburgh, PA, USA) at 4% for 2 min; dye was done by aspiration, and the wells were washed twice with 100 μL of 1× PBS. The presence or absence of color was observed. Subsequently, the CV of each well was removed with 100 μL of alcohol: acetone solution 80:20 (*v*/*v*), adjusted to a final volume of 2 mL with the same solution; the biomass was quantified with spectrophotometric reading using a plate reader (BioRad iMark, Hercules, CA, USA) at 570 nm [34]. Each assay was performed in triplicate. Strains with an absorbance value < 0.001 were classified as null biofilm producers, values between 0.001 and 0.500 were weak biofilm producers, absorbance values of 0.501–0.900 were moderate biofilm producers, while those with absorbance values ≥ 0.901 were considered as high biofilm producers [36].

### 2.5. Genotypic Determination of mecA, icaADBC, and bap

The molecular detection of the methicillin resistance was conducted by the amplification of the *mecA* gene. To determine this gene and its relation to biofilm production, the gDNA of the isolated *S. aureus* strains was obtained by the guanidine method [34]. DNA integrity was determined by 2% agarose gel electrophoresis (1× TBE buffer at 150 V for 30 min), and purity was determined using Nanodrop equipment (Thermo Scientific, Waltham, MA, USA) through the relationship of absorbances at 260/280 nm. *S. aureus* ATCC 43300 was used as a positive control of the *mecA* gene, *S. aureus* ATCC 25923 was used as a negative control, and *S. epidermidis* ATCC 12228 was used as a positive control for the detection of *icaADBC*+ genes [34]. *S. aureus* ATCC 29247 was used as a negative control [37].

Six genes were amplified for the detection of biofilm formation of hospital-acquired strains. The primer sequences are presented in Table 1 [34,38]. The reaction mixture for gene amplification was: 2.5 μL of dNTP (2.5 mM), 2.5 μL of PCR buffer, 2 μL of MgCl_2_ solution (1.5 mM), 1 μL of required F and R primer (10 pmol), 1 µL of DNA solution (50 ng/µL), 0.2 µL (1 U/µL) of Taq polymerase (Thermo Scientific, USA), and nuclease-free water for a final mixture of 25 µL. The gene amplifications were carried out according to what was indicated by Martins et al., 2017, with some modifications. Initial denaturation at 94 °C/5 min for 30 cycles: denaturation at 94 °C/30 s, alignment 50 °C/30 s (*icaA*), 54 °C/60 s (*icaBCD*), 55 °C/30 s (*mecA*), and 56 °C/30 s (*bap*), and extension 72 °C/10 min (*icaA* and *icaBCD*) and 72 °C/5 min (*mecA* and *bap*), followed by a final extension at 72 °C/10 min [34,39].

The genotypic determination of the staphylococcal cassette chromosome *mec* (SCC*mec*) was carried out.

The amplification of the SCC*mec* genes associated with methicillin resistance was carried out from bacterial *g*DNA, using the oligonucleotides in Table 2 [40].

For the SCC*mec* type identification, two groups were formed, the first one to identify types I, II, and III that correspond to healthcare-associated strains and the second to identify types IVa, IVb, IVc, IVd, and V that correspond to strains acquired in the community. In the first case, the mix contained water 9.5 μL; F and R of oligonucleotides type I, II, and III, 0.5 μL (0.2 pmol); PCR Master Mix (BioRad; CA, USA) 12.5 μL; and gDNA 2 μL (100 ng/μL). Amplification conditions for simple PCR for types I, II, and III were carried out according to what was indicated by Zhang et al. (2005) [40], with some modifications: one cycle of initial denaturation at 94 °C/5 min, 30 cycles of denaturation at 94 °C/1 min, annealing at 50 °C/1 min, and extension at 72 °C/2 min, followed by a final extension at 72 °C/10 min.

The second mix contained water 2 μL; primer F and R of oligonucleotides type V, type IVa, type IVb, type IVc, and type IVd, 1 μL; PCR Master Mix (BioRad, USA) 11 μL; and *g*DNA 2 μL (100 ng/μL). Amplification conditions for simple PCR for types IVa, IVb, IVc, IVd, and V were carried out according to what was indicated by Zhang et al. 2005 [40], with some modifications: one initial denaturation cycle at 94 °C/5 min, 10 denaturation cycles 94 °C/45 s, alignment 65 °C/45 s, extension 72 °C/90 s, 25 denaturation cycles 94 °C/45 s, alignment 55 °C/45 s, extension 72 °C/90 s, followed by a final extension at 72 °C/1 min. For the SCC*mec* gene, *S. aureus* RM911 (for SCC*mec* types I), *S. aureus* RM912 (for SCC*mec* II), *S. aureus* RM913 (for SCCmec types III), *S. aureus* RM914 (for SCC*mec* IV) was used as a positive control, and *S. aureus* RM917 (for SCC*mec* V) and sterile DNase-free water were included in each PCR as a negative control [41].

### 2.6. Statistical Analysis

The relationship between resistance to methicillin and biofilm formation was determined using the Student’s t statistical test.

## 3. Results

### 3.1. Strain Isolation and Identification

A total of 80 healthcare-associated Gram-positive cocci from the third level belonged to the *Staphylococcus* genera; among them, six different species were identified, 46% (37/80) belonged to *S. aureus* and 54% (43/80) to the SOSA species. We considered SOSA as all other non-aureus strains due to the coagulase-variable nature of some species [42]. A total of 35 SOSA strains belonged to *S. epidermidis*, 4 to *S. haemolyticus*, 2 to *S. hominis*, 1 to *S. intermedius*, and 1 to *S. saprophyticus*. In total 56.25% of the strains were isolated from the blood culture, 28.75% from the catheter, 6.25% from the cerebrospinal fluid, 6.25% from secretions of the respiratory tract, and 2.5% from urine. *S. epidermidis* could cause multidrug-resistant infection in immunocompromised patients, bacterial sepsis, foreign body-related infections, and biofilm-associated infections [43,44]. *S. haemolyticus* could cause severe infections like meningitis, endocarditis, prosthetic joint infections, bacteremia, septicemia, peritonitis, and otitis generally in immunocompromised patients and animals [45]. *S. hominis* is rarely a human pathogen, which could cause soft tissue infections and bacteremia in hospitalized patients [46]. *S. intermedius* is an animal pathogen and could be a pathogen in oncology for human patients [47]. *S. saprophyticus* is a urinary tract infection pathogen and in a few cases, bacteremia [48].

### 3.2. Antibiotic Resistance

A total amount of 85% (68/80) of *Staphylococcus* spp. were resistant to cefoxitin, 86% (32/37) of the strains were MRSA, and 84% (36/43) were MRSOSA (Table 3). Molecular surveillance of MRSA clones is important to understand their evolutionary dynamics for investigating outbreaks, propagating precautionary measures, as well as planning for appropriate treatment [49]. Additionally, the multidrug resistance complicates the treatment of patients infected with MRSA and MRSOSA strains; therefore, knowing the antibiotic pattern resistance could lead to a diminished hospital outbreak, dissemination of multidrug strains, mortality rates, and hospitalization cost and time.

Finally, 14% (5/37) were SA no MR and 16% (7/43) were SOSA no MR. The percentage of resistance by all species different to SA is shown in Table 4.

All strains isolated from the *Staphylococcus* genus were susceptible to vancomycin.

### 3.3. β-Lactamase Production

The presence of the enzyme was observed in 74% of the total strains (59/80); 54% (32/59) corresponded to MRSA strains, while 46% (27/59) corresponded to MRSOSA. A total of 86% (32/37) of *S. aureus* isolated strains, while only 63% (27/43) of SOSA, were producers of *β*-lactamases. MR β-lactamase producers are shown in Table 3. For each SOSA species, the percentage of β-lactamase production is shown in Table 4.

### 3.4. Determination of Biofilm Production

In general, 100% of the strains developed biofilm formation. Weak, moderate, and high production of *S. aureus* strains and SOSA are shown in Table 3. For each SOSA species, the percentage of biofilm production is shown in Table 4. *S. aureus* colonizes tissue surfaces in humans, causing chronic persistent infections that are difficult to cure due to its antibiotic recalcitrance and phenotypic adaptability, both of which are facilitated by its ability to develop biofilms [50]; thus, it is essential to develop strategies aimed at avoiding the production of biofilm by *S. aureus* and SOSA.

### 3.5. Genotypic Determination of mecA and SCCmec Types

The *mecA* gene of all strains amplified 310 bp [51]; 85% (68/80) of the strains were MR (Table 3). However, only 65 strains out of 68 amplified *mecA*, of which 84% (31/37) of the MR strains belonged to *S. aureus* and 79% (34/43) to other species. A total of 59 out of 68 MR strains were *β*-lactamase producers (Table 3), suggesting that *Staphylococcus* may have more than one resistance mechanism. Table 3 also shows the frequency of the types of SSC*mec*. The majority presented type II (41.54%, 27/65) and III (30.7%, 20/65) (Table 3), which confirms that most of the isolates came from healthcare-associated infections. For type V, no positive strains were obtained. Thus, 73.8% (48/65) of strains were classified as healthcare-associated infections; the rest were acquired in the community. It has been reported that SCC*mec* types II and III that exhibit multi-resistance also have genes for erythromycin and tetracycline resistance, while community-acquired MRSA strains (CA-MRSA) produce more virulent infections and infect healthy people outside of hospitals [52]. Strains of *S. hominis*, *S. epidermidis*, and *S. aureus* type IV related to CA could have zoonotic transmission dynamics; however, in this study, it was not investigated.

From that percentage, 70.9% (22/31) belonged to SA and 76.4% (26/34) to SOSA. In the case of the strains acquired in the community, 16.12% (5/31) were SA and 14.7% (5/34) were SOSA. Seven *mecA* positive strains did not amplify for any SCC*mec* type, from which four and three were MRSA and MRSOSA, respectively. Table 4 shows the expression of *mecA* and SSC*mec* genes, regarding the different percentage of biofilm and *β*-lactamase production, and MR to the different SOSA species.

### 3.6. Genotypic Determination of icaADBC and bap Genes

In general, it was found that 47.5% (38/80), 32.5% (26/80), 18.75% (15/80), and 41.25% (33/80) of the strains presented the genes *icaA*, *icaB*, *icaC*, and *icaD*, respectively. For strains of the genus *aureus*, an amplification ratio of 51.3% (19/37), 32.4% (12/37), 24.3% (9/37), and 43.24% (16/37) was found, and for SOSA strains, 44.1% (19/43) and 32.5% (14/43) were found, respectively. Table 3 shows the percentage of each gene of the *ica* operon by SA or SOSA. Table 4 shows the expression of the *icaABCD* gene, regarding the different types of SSC*mec*, expression of *mecA*, biofilm and β-lactamase production, and MR to the different SOSA species. Any of the strains amplified the *bap* gene.

The molecular size detected for each fragment gene were 319 bp for *icaA*; 409 bp for *icaB*; 148 bp for *icaC*, and 150 bp for *icaD*, which correspond with Zhang et al. (2005) [40].

Bap promotes adhesion to abiotic surfaces and induces strong intercellular adhesion by self-assembling into amyloid-like aggregates in response to the levels of calcium and pH in the environment. During infection, *Bap* enhances the adhesion to epithelial cells where it binds directly to the host receptor Gp96 and inhibits the entry of the bacteria into the cells [53]. Bap has been reported in bovine mastitis isolates of *S. aureus* and their absence in human clinical isolates, since Bap-mediated biofilm seems to be a system specialized for the conditions present in the mammary gland, where calcium concentration can reach the high values necessary to modulate *Bap* function (~10 mM). Thus, calcium serves as a regulator of *Bap* function; the fluctuations in the local calcium concentration should be higher than the binding affinity of the protein for the cation [54].

## 4. Discussion

According to the Pan American Health Organization (2021), *Saphylococcus aureus* is one of the microorganisms that has shown higher levels of resistance to various generations of antibiotics in recent times, becoming a public health problem classified as an “urgent health problem of global dimension” (PHAO, 2021). In fact, MRSA infections are one of the most serious multidrug-resistant threats, require longer hospitalization times, can represent up to 80% of healthcare-associated infections [41], and have higher mortality rates [55]. The WHO has suggested that people with methicillin-resistant *S. aureus* (MRSA) infections are 64% more likely to die than people with drug-sensitive infections (WHO, 2021). In Mexico, according to RHOVE (Bulletin of Infections Associated with Health Care Hospital Epidemiological Surveillance Network) 2022, *S. aureus* is the number five microorganism associated with healthcare-associated infections, with 2091 HAI reported in 2022. In 2023, RHOVE for the first trimester reported *S. aureus* as the number five microorganism that produces healthcare-associated infections in Mexico; in the second trimester, *S. epidermidis* was reported as number five, which represents almost 30% of infections acquired in Mexico hospitals [41].

In this study, 80 strains of different clinical origin were isolated from a tertiary public hospital in Mexico City, most of them confirmed as *S. aureus* and *S. epidermidis* (46% and 44%, respectively). The remaining strains represented 10% (8/80) and belonged to the *haemolyticus*, *hominis*, *intermedius*, and *saprophyticus* species. Negrete-González et al. (2020) [8] reported the isolation of 191 *S. aureus* from the emergency department, surgery, intensive care unit, internal medicine, gynecology, burn unit, and outpatient service from a hospital ubicated in San Luis Potosi, Mexico. López-Jácome et al. (2020) [56] reported the isolation of 96 strains from electric-burned patients in a referral burn hospital in Mexico City. Also, *S. aureus* MRSA has been reported in fishermen and horticulturists [57] from Guerrero, Mexico. On the other hand, *S. epidermidis* has been reported as a relevant microorganism based on its high ability to develop biofilm and small colony variants [58]. In Mexico, it has been isolated from children’s hospitals [59]. Martinez- Santos et al. (2022) [60] isolated 20 methicillin-resistant *S. epidermidis* from hospitalized patients with bloodstream infections from two different hospitals from Acapulco, Guerrero México, between 2003 and 2004, and 2017. Fernández-Rodríguez et al. (2021) [58] isolated *S. epidermidis* and variants of this microorganism from patients with monomicrobial prosthetic joint infection at Instituto Nacional de Rehabilitacion “Luis Guillermo Ibarra Ibarra” in Mexico City. This microorganism has also been isolated from feedings and feces of preterm neonates in Spain [61]. Globally, three multidrug-resistant, hospital-adapted lineages of *S. epidermidis* (two ST2 and one ST23) have emerged in recent decades and disseminated [62]. In the present work, it was found that *S. aureus* and *S. epidermidis* were the most abundant (37/80 and 35/80), which is relevant since these strains are MR and less susceptible to glycopeptides, which complicate the treatment, as has been suggested by Becker et al. (2014) [63] in other clones.

*S. haemolyticus* have been isolated from clinical samples [64] and *S. hominis* from joint prosthesis, periprosthetic tissue, joint fluids, and fluid sonication of the joint prosthesis of the hip and knee from Mexican patients in Guadalajara and Nuevo León [65]. *S. hominis* have also been isolated from the blood [66] of patients with surgical-site infections undergoing cardiovascular surgery through median sternotomy [67]. *S. intermedius* have been isolated from hepatic abscess in a patient from Mexico City [68].

One of the limitations of our study is that the collection of the strains cannot be described regarding the number of copy strains excluded, the duration, and the percentages of isolation, since they were kindly donated by the clinical analysis laboratory of different hospitals of the public sector in Mexico.

In 2021, Chen et al. [69] indicated that community-associated MRSA (CA-MRSA) has replaced HA-MRSA as the dominant epidemic strain, among which the *Staphylococcus* genus is found, which is consistent with our study, where we found 73.8% HA-MRSA and 26.15% CA-MRSA.

One of the main factors of resistance to glycopeptides is biofilm production, a mechanism used among the *Staphylococcus* group as a virulence factor [70]. The use of this strategy by the isolated strains was observed in our study, where 100% of the strains were biofilm producers with a weak, moderate, and high severity, of which 46.2% corresponded to *S. aureus* (24.3, 45.9, and 29.72 severity, respectively) and the 53.8% to SOSA (9.3, 39.5, and 51.11 severity, respectively), which could favor its resistance to antibiotics. The observed response is not different due to the origin of the sample; this may be due to a modification of the biofilm by regulatory mechanisms, depending on the environmental conditions or exposure to antibiotics, which increases resistance to them and even in the host immune defenses [71]. The *icaABCD* operation in isolates of *S. aureus* was associated with the formation of biofilms. Ghaioumy and col. (2021) [72] reported that from 46 clinical samples, 41% expressed *icaA* and *icaD* (6.3% and 59.4%, respectively), although *icaC* and *icaB* were not detected, and 100% of the isolates of *S. aureus* were biofilm producers. In our study, the expression of the genes *icaA*, *icaB*, *icaC*, and *icaD* (51.3%, 32.4%, 24.3%, and 43.24%, respectively), were detected in *S. aureus*, while in SOSA strains, the genes *icaA* and *icaB* (44.1% and 32.5%) were detected, and all the strains were biofilm producers. A study carried out by García et al. (2019) [34] at the Instituto Nacional de Rehabilitacion in Mexico City, with clinical samples of hospitalized patients, reported an expression of the *icaADBC* genes in *S. aureus* and *Staphylococcus* coagulase negative strains (91% and 92%, respectively), which was associated with the biofilm production in 100% of the strains.

It has been shown that some SOSA strains can produce biofilm through the expression of genes *ica*, *Aap*, and *bap*; the latter was reviewed for the strains of this study, but no strain was amplified; this may be because the strains in which it has been reported have been from veterinary studies, and those shown here are from clinical isolates, or because biofilm production is due to other genes not described in this study [73,74,75,76,77].

An important element in biofilm formation and one of the most studied, is the *ica* operon, a group of genes that encodes the production of PIA/PNGA, which mediates intercellular adhesion of bacteria and biofilm accumulation. However, in various studies, only *icaA* and *icaD* were amplified. In these positive strains, there was PIA formation; however, it has been evaluated that the presence of the entire operon acts together to increase biofilm production [74,76,77]. As mentioned, biosynthesis genes clustered in the *ica* operon contribute to the formation of biofilms; they have been identified in *S. epidermidis*, *S. aureus*, *Bacillus subtilis*, and in many Gram-negative bacteria [78]. Expression of the *icaADBC* operon appears tightly controlled in *S. aureus*, as evidenced by the fact that it is expressed at very low levels under in vitro growth conditions [78].

In our work, the percentage of detection of the *icaADBC* genes varied among strains, probably due to the arrangement of the operon, since it can form forks and thus interrupt transcription; it may also be incomplete due to a mutation in some part of it. In addition, biofilm production could be due to other mechanisms or genes not studied in this work. The amplification percentages of *icaADBC* genes *S. aureus* isolates were slightly increased with respect to SOSA. We expected that all the strains that showed greater biofilm production would present the complete *ica* operon; however, the detection of the *ica* operon was not possible in all strains. This may be because there are other genes involved in biofilm formation, such as the *Aap*; others related to a large group of receptor proteins (MSCRAMM) involved in the adhesion mechanism of the microorganism to the extracellular matrix of the host; the CcpA protein that has an important impact on the regulation of the operon, which in turn is involved in the synthesis of PIA; the SasG surface-associated *Staphylococcus* protein G; and other genes related to the production of polymers associated to biofilms such as extracellular teichoic acid [79].

Although the *ica* operon has been the most studied, in the literature, it is found that of the strains that produce biofilm, only 30% present high levels of PIA in vitro; the fact that PIA is not detected, could be present at levels not detectable or even absent, may suggests that the biofilms are composed mainly of teichoic acid and other protein components [74,79].

It has been reported that the resistance of *S. aureus* to β-lactam antibiotics is controlled by the BlaR13 receptor that senses β-lactams through acylation of its sensor domain, inducing transmembrane signaling and activation of the cytoplasm-oriented metalloprotease domain. This domain induces the expression of *blaZ* (β-lactamase PC1) and *mecA* (β-lactam-resistant cell wall transpeptidase PBP2a) [80], the latter encoding the alternative penicillin-binding protein, PBP2A, which is insensitive to antibiotics. Another reason for resistance is due to additional genetic adjustments to develop a high level of resistance [81]. Methicillin is a semisynthetic β-lactam resistant to β-lactamase. Antibiotic resistance by *S. aureus* has spread in epidemic waves, being a MRSA healthcare- associated infection, giving rise to HA-MRSA. More recently, CA-MRSA has emerged as a major clinical threat, creating a reservoir of MRSA within and outside of healthcare settings. CA-MRSA can be genetically distinguished from HA-MRSA, as it also has fewer antibiotic resistance properties and often produces the toxin Panton–Valentine leukocidin (PVL). However, there are now many examples of how CA-MRSA has spread to healthcare settings, blurring the distinction between the two types of MRSA. Additionally, MRSA can be harbored by livestock (livestock-associated MRSA [LA-MRSA]), where it can cause disease in those animals and be transmitted to humans through contact [81]. Another resistance mechanism is the secretion of β-lactamase enzymes that are encoded by mobile elements that are transferable between species, as is the case of the *mecA* gene that encodes the production of the penicillin-binding protein (PBP). This is the most common mechanism in strains of *S. aureus* and MRSA [82], which is consistent with our study, where we observed that 74% of the total strains were secretors of β-lactamase, from which 54% were MRSA and 46% MRSOSA, related to the 100% frequency of the resistance to methicillin of the strains isolated in our study. This resistance phenotype was like that reported by García et al. 2019 [34], where they found the *mecA* gene in 78% of the total clinical samples analyzed. On the contrary, the expression of the gene in our study was higher than that reported by Hashem et al. 2017 [83], where it is only expressed in 45% of *S. aureus* strains, 35% *S. epidermidis*, and 16.7% in other Staphylococcus species isolated from catheters. The CLSI recommends corroborating methicillin resistance with the detection of the *mecA* gene. Some authors mention that the detection of this gene is considered the gold standard, since it agrees with the disk diffusion test by 90%, as determined in this study [84]. It is worth mentioning that there are other resistance mechanisms not studied in this work that may also intervene and need further research.

The amplification of the SSC*mec* types was also carried out as a mobile element inserted in the chromosome of MRSA and MRSOSA that contains the set of *mec* genes, corresponding to the *mecA* genes and their regulators. The amplification was carried out in 65 of the 80 strains that amplified *mecA*; the majority presented type II, 41.54% (27/65), followed by type III, 30.77% (20/65), and type IV 15.38% (10/65); the amplified types were mostly IVa, with only one type IVc strain, and less frequently, type I representing 1.54% (1/65). Eight well-identified types of SCC*mec* were obtained. It is important to mention that types I, II, and III of SCC*mec* were HA-MRSA strains. However, since they are relatively large chromosomal “cassettes,” these allow a greater number of resistance genes to be housed for other antimicrobial agents; for this reason, healthcare-associated strains have greater resistance to several antimicrobials compared to those acquired in the community [40,78,85]. The *SCC* type of *mec*, where CA-MRSA with the smaller type IV element can maintain a growth rate and toxin production levels in vitro, compared to HA-MRSA with the larger type II, suggests a role in the ability of strains to compete in the community environment. CA-MRSA has evolved several times, where an evolutionary trade-off has been achieved between maintaining antibiotic resistance and enhanced pathogenicity, but without sacrificing overall fitness [81].

## 5. Conclusions

It was observed that 85% of the isolated strains (68/80) were resistant to methicillin, 95.58% (65/68) presented the *mecA* gene, and 86.77% (59/68) were β-lactamase producers. Methicillin resistance was mainly mediated by the *mecA* gene; however, there may be other regulatory mechanisms, such as the genes Mec1 and Bla1, that more effectively control the expression of *mecA*; Bla1 is more efficient because Mec1 is not present in MRSA clinical isolates. The Mec1 repressor and transmembrane MecR1 sensor protein regulate PBP2a synthesis, a penicillin-binding protein that has taken the place of the PBP, which is responsible for the cross-binding of peptidoglycan in MRSA, as has been suggested by Alghamdi et al. (2023) [52]; *mecA* and *mecC* genes may also originate in coagulase-negative staphylococci. In our study, 73.84% (48/65) of the isolated strains were hospital-acquired methicillin-resistant strains, with 48.14% (27/65) belonging to Type II. Another form of mec-independent resistance in clinical samples is MODSA (modified penicillin-binding protein *S. aureus)* strains, which have mutations in PBP2 and other PBPs present in the transpeptidase domain targeted by β-lactams; their level of resistance is low compared to that of *mecA* MRSA [86], which is a possible explanation not studied in this work. In the studied population, no vancomycin-resistant *S. aureus* strains were found, which reduces the risk of mortality, considering that this microorganism is a devastating agent due to its resistance to multiple drugs.

In this study, methicillin resistance was mainly mediated by the *mecA* gene; however, there may be other mechanisms that also participate, since biofilm production is related to genes of the *icaADBC* operon, and methicillin resistance was not associated with biofilm production. Therefore, it is necessary to strengthen surveillance to prevent the spread of these outbreaks both in the nosocomial environment and the community. MRSA isolates usually have higher biofilm-production ability, as MRSA *mecA* gene encodes PBP2a and inactivates the *agr* gene quorum-sensing regulator system, thereby enhancing biofilm formation, as has been suggested by Maharjan et al. (2022) [87]. In addition, this ability is specific to each strain and associated with different environmental conditions; it causes great resistance to the action of various antimicrobial agents, leading to persistence and recurrence of infections. Thus, other authors have suggested treatment with dispersion-enzyme B to reduce biofilm production in clinical MRSA strains [87].

## Figures and Tables

**Table 1:** *icaADBC*, *mecA*, and *bap* oligonucleotide sequences and thermal conditions [34,39].

Gene	Sequence (5′-3′)	Annealing Temperature °C	PCR Product Size (bp)
*icaA*	F: CGTTGATCAAGATGCACC	59.2	319
	R: CCGCTTGCCATGTGTTG	60.9	
*icaB*	F: TGGATTAACTTTGATGATATGG	54.3	409
	R: AGGAAAAAGCTGTCACACC	55.3	
*icaC*	F: GGTCAATGGTATGGCTATTT	54.1	148
	R: CGAACAACACAGCGTTTC	56.2	
*icaD*	F: GGTCAAGCCCAGACAGAG	56.7	150
	R: GAAATTCATGACGAAAGTATC	54.3	
*mecA*	F: TGGCTATCGTGTCACAATCG	60.3	310
	R: CTGGAACTTGTTGAGCAGAG	59.7	
*bap*	F: GGCGATGGTAAGAATGATGG	60.3	515
	R: GCTGTTGAAGTTAATACTGTACCTGC	59.7	

**Table 2:** SCC*mec* type oligonucleotide sequences.

Gene Type	Sequence (5′-3′)	Amplicon Size (bp)	Specificity
I	F: GCTTTAAAGAGTGTCGTTACAGG	613	SCC*mec* I
	R: GTTCTCTCATAGTATGACGTCC		
II	F: CGTTGAAGATGATGAAGCG	398	SCC*mec* II
	R: CGAAATCAATGGTTAATGGACC		
III	F: CCATATTGTGTACGATGCG	280	SCC*mec* III
	R: CCTTAGTTGTCGTAACAGATCG		
IVa	F: GCCTTATTCGAAGAAACCG	776	SCC*mec* IVa
	R: CTACTCTTCTGAAAAGCGTCG		
IVb	F: TCTGGAATTACTTCAGCTGC	493	SCC*mec* IVb
	R: AAACAATATTGCTCTCCCTC		
IVc	F: ACAATATTTGTATTATCGGAGAGC	200	SCC*mec* IVc
	R: TTGGTATGAGGTATTGCTGG		
IVd	F: CTCAAAATACGGACCCCAATACA	881	SCC*mec* IVd
	R: TGCTCCAGTAATTGCTAAAG		
V	F: GAACATTGTTACTTAAATGAGCG	325	SCC*mec* V
	R: TGAAAGTTGTACCCTTGACACC		

**Table 3:** Relationship of biofilm production in isolated strains and methicillin resistance, detection of *β*-lactamases, amplification of the *mecA* gene, and types of genes belonging to SCC*mec* in *S. aureus* and SOSA.

				Biofilm Production				SCC*mec* Type			
	MR % n	β-Lactamase % n	*mecA* % n	Low % n	Moderate % n	High % n	Total % n	I % n	II % n	III % n	IV % n
*S. aureus*	86	100	96.8	24.3	45.9	29.72	46.20%	3.22	45.16	22.5	16.1
	32/37	32/32	31/32	9/37	17/37	11/37	37/80	1/31	14/31	7/31	5/31
					*ica* %/n						
A				44.44	52.94	54.55					
				4/9	9/17	6/11					
B				44.44	47.06	54.55					
				2/9	6/17	4/11					
C				22.22	35.29	36.36					
				1/9	4/17	3/11					
D				11.11	23.53	27.27					
				4/9	8/17	6/11					
SOSA	83.7	75	94.4	9.3	39.5	51.11	53.80%	0	38.23	38.23	14.7
	36/43	27/36	34/36	4/43	17/43	22/43	43/80	0/34	13/34	13/34	5/34
					*ica* %/n						
A				25	47.06	50					
				1/4	8/17	11/22					
B				25	35.29	45.44					
				1/4	5/17	8/22					
C				25	29.41	36.36					
				1/4	0/17	5/22					
D				25	0	27.73					
				1/4	6/17	10/22					
Total	85	86.7	95.5	16.2	42.5	41.2	1	1.54	41.54	30.7	15.4
	68/80	59/68	65/68	13/80	34/80	33/80	80/80	1/65	27/65	20/65	10/65

**Table 4:** Relationship of biofilm production in isolated strains and methicillin resistance, detection of *β*-lactamases, amplification of the *mecA* gene, and types of genes belonging to SCC*mec* in SOSA.

Strain	MR	β-Lactamase	Biofilm	*mecA*	SSC*mec*	*icaA*	*icaB*	*icaC*	*icaD*
*S. epidermidis* (35/80) 44%	Resistance (28/35) 80%	NS (8/28) 23%	High (4/8) 50%	+	NA	−	−	−	−
					Type III	−	−	−	−
						+	+	+	+
			Moderate (3/8) 38%	+	Type II	−	−	−	−
					Type III	+	−	−	−
					Type III	−	−	−	−
			Low (1/8) 13%	+	Type III	+	+	+	+
		S (20/28) 57%	High (12/20) 60%	−	NA	+	+	−	+
				+	Type III	−	−	−	−
					Type II	−	−	−	−
						+	−	−	+
						+	+	+	+
					Type III	−	−	−	−
						+	−	−	+
						+	+	+	+
					Type IV	−	−	−	−
			Moderate (7/20) 35%	+	Type II	−	−	−	−
						+	+	−	+
					Type III	−	−	−	−
						+	+	−	+
					Type IV	−	−	−	−
						+	+	−	+
			Low (1/20) 5%	+	Type II	−	−	−	−
	Susceptible (7/35) 20%	NS (6/7) 17%	High (2/6) 33%	−	NA	−	−	−	−
						+	+	−	+
			Moderate (2/6) 33%	−	NA	−	−	−	−
			Low (2/6) 33%	−	NA	−	−	−	−
		S (1/7) 3%	Moderate (1/1) 100%	−	NA	+	−	−	−
*S. haemolyticus* (4/80) 5%	Resistance (4/4) 100%	NS (1/4) 25%	High (1/1) 100%	+	Type III	+	+	+	+
		S (3/4) 75%	High (1/3) 33%	+	Type II	−	−	−	−
			Moderate (2/3) 67%	+	Type II	−	−	−	−
						+	−	−	+
*S. hominis* (2/80) 3%	Resistance (2/2) 100%	S (2/2) 100%	High (1/2) 50%	+	Type IV	+	+	+	+
			Moderate (1/2) 50%	+	Type IV	−	−	−	−
*S. intermedius* (1/80) 1%	Resistance (1/1) 100%	NS (1/1) 100%	High (1/1) 100%	+	Type II	−	−	−	−
*S. saprophyticus* (1/80) 1%	Resistance (1/1) 100%	S (1/1) 100%	Moderate (1/1) 100%	+	Type II	+	+	−	+

“−” indicates absence of the gene; “+” indicates presence of the gene.

## Data Availability

Data are contained within the article.
